# Mitochondria-Targeted Antioxidants Prevent Tachypacing-Induced Contractile Dysfunction in In Vitro Cardiomyocyte and In Vivo *Drosophila* Models of Atrial Fibrillation

**DOI:** 10.3390/antiox14121444

**Published:** 2025-11-30

**Authors:** Alexia van Rinsum, Liangyu Hu, Xi Qi, Jaap Keijer, Deli Zhang

**Affiliations:** Human and Animal Physiology, Wageningen University and Research, 6708 WD Wageningen, The Netherlands; alexia.vanrinsum@wur.nl (A.v.R.); liangyu.hu@wur.nl (L.H.); xi.qi@wur.nl (X.Q.); jaap.keijer@wur.nl (J.K.)

**Keywords:** mitochondrial dysfunction, antioxidant, cardiac arrhythmia, *Drosophila*, iAM, MitoTEMPO, Sul-238, mitochondrial oxidative stress, atrial fibrillation

## Abstract

Atrial fibrillation (AF) is a growing cardiovascular epidemic lacking effective treatment. Reactive oxygen species are believed to contribute to AF pathophysiology, yet general antioxidants have limited effectiveness. Since mitochondria are abundant in the heart and a major ROS producer, mitochondrial oxidative stress (MitoOxS) could be a therapeutic target. To determine this, rat inducible immortalized atrial myocytes (iAMs) were tachypaced to mimic AF, followed by assessment of calcium transients, contractility, mitochondrial respiration and morphology, and ROS damage. Cells were pretreated with MitoTEMPO or Sul-238 to assess their protective effects. In *Drosophila*, heart wall contractions were analyzed to assess arrhythmogenesis after mitochondrial antioxidant pretreatment. Using the GAL4-UAS system, mitochondrial ROS levels and the effect of SOD1 or SOD2 knockdown or overexpression on arrhythmogenesis were evaluated. Tachypacing induced contractile dysfunction and arrhythmogenesis, mitochondrial impairment, and ROS damage in iAMs and increased mitochondrial ROS and arrhythmogenesis in *Drosophila*. Both MitoTEMPO and Sul-238 treatments prevented mitochondrial dysfunction and arrhythmogenesis in iAMs and rescued arrhythmia in *Drosophila*. Underscoring the potential to target MitoOxS specifically, SOD2 knockdown promoted arrhythmogenesis in iAMs and *Drosophila*, whereas SOD2 overexpression rescued tachypacing-induced arrhythmia. MitoOxS is thus a key driver of tachypacing-induced contractile dysfunction and arrhythmia. Mitochondria-targeted antioxidants, such as MitoTEMPO or Sul-238, represent promising therapeutic strategies for AF.

## 1. Introduction

Being the most common clinically significant cardiac rhythm disorder, atrial fibrillation (AF), is considered a 21st-century cardiovascular disease epidemic [1]. Importantly, over 98% of AF is caused by risk factors, including ageing, cardiac diseases, metabolic syndrome, and diabetes mellitus [2]. With the global population aging and the occurrence of metabolic disorders on the rise, the prevalence of individuals with AF is expected to rise even further, to 15.9 million in America by 2050 [3] and 17.9 million in Europe by 2060 [4]. Without proper treatment, this is likely to lead to an even greater socioeconomic burden; yet the precise mechanisms driving AF development are still not fully understood.

The pathophysiology of AF involves a large number of significant players, including reactive oxygen species (ROS) generation and oxidative stress [5,6]. Oxidative stress arises when ROS production exceeds the capacity of the cellular antioxidant system. Since oxidative stress is indicated in the pathophysiology of AF, it is not surprising that treatment of AF patients with antioxidants has been investigated. Unfortunately, clinical trials using general antioxidant therapies for AF have not been met with much success [7,8]. This suggests that general antioxidant therapies do not target the pathogenic source of oxidative stress. The heart has a high energy demand and is therefore rich in mitochondria. As the primary site for oxidative phosphorylation, mitochondria naturally produce ROS as a byproduct of adenosine triphosphate (ATP) generation. Importantly, increased mitochondrial ROS production, without adequate antioxidant response, leading to mitochondrial oxidative stress (MitoOxS) and mitochondrial dysfunction, has been implicated in many of the risk factors associated with AF [9]. Consequently, MitoOxS has emerged as a potential therapeutic target for AF.

Several mitochondria-targeted antioxidants have been developed to specifically counteract MitoOxS for a wide range of diseases. Examples of these are the established MitoTEMPO and the recently developed Sul-238 compound. MitoTEMPO is a derivative of the antioxidant piperidine nitroxide (TEMPO) conjugated to a lipophilic triphenylphosphonium (TPP+) cation [10]. The TPP+ is a membrane-permeant cation that accumulates several hundredfold within mitochondria, while TEMPO acts as a superoxide dismutase (SOD) mimetic, converting superoxide (O_2_^−^) into hydrogen peroxide (H_2_O_2_) and oxygen (O_2_) [10,11]. The Sul-238 is a structural derivative of 6-chromanol, the core scaffold of tocopherols (vitamin E) and Trolox [12]. Sul-238 has been reported to support mitochondrial function by prevention of reverse electron flux, thereby reducing mitochondrial ROS production and maintaining mitochondrial ATP production in several preclinical models [13,14,15].

SODs are a group of enzymes that play a crucial role in the ROS detoxification and maintenance of cellular redox balance. Three SODs have been discovered, of which SOD1 is primarily located in the cytosol and is sparsely present in the mitochondrial innermembrane space, SOD2 is primarily located in the mitochondrial matrix, and SOD3 is primarily located extracellularly [16].

In this study, we first determined the effect of pretreatment with MitoTEMPO or Sul-238 on contractile function, mitochondrial respiration, and ROS-induced lipid peroxidation and DNA damage in an induced immortalized atrial myocyte (iAM) cellular model of AF. We found that tachypacing induces contractile dysfunction, decreased mitochondrial respiration and increased ROS-damage, which was prevented by both MitoTEMPO, as well as Sul-238 treatment. These results were confirmed in an in vivo *Drosophila* melanogaster model of AF. Lastly, we validated that the increase in MitoOxS specifically induces the contractile dysfunction and increased arrhythmia in iAMs and *Drosophila* by knocking down SOD1 or SOD2. Together, the results show that increased MitoOxS induces contractile dysfunction, which is associated with mitochondrial dysfunction and ROS-induced damage, underscoring the potential of targeting MitoOxS to prevent AF development.

## 2. Materials and Methods

### 2.1. Cell Culture

Proliferating iAMs (colony 2.16), derived from neonatal rat atria by Liu et al. [17], were maintained in proliferation medium, consisting of advanced Dulbecco’s modified Eagle’s medium/F12 (DMEM/F-12, Thermo Fisher, Waltham, MA, USA, #12634010) supplemented with 2% fetal bovine serum (FBS, Thermo Fisher, #10500064), 1× GlutaMax (Thermo Fisher, #35050038), 50 μg/mL penicillin–streptomycin (PenStrep, Thermo Fisher, #15140122), and 100 ng/mL doxycycline (Sigma Aldrich, St. Louis, MO, USA, #D9891). The proliferating iAMs were maintained in the logarithmic phase. Upon reaching 90% confluency, living cells were passaged in a 1:3 ratio to a new culture flask for further multiplication or assaying. To this end, the cells were washed twice using Dulbecco’s phosphate-buffered saline (DPBS, Thermo Fisher, #14190144) and dissociated with 0.05% trypsin-EDTA (Thermo Fisher, #15400054) for 3 min. Cells were then centrifuged at 100–175× *g* for 5 min at room temperature (RT) and subsequently resuspended in proliferation medium to continue culturing or in differentiation medium for experiments. Differentiation medium consisted of advanced DMEM/F12 medium supplemented with 2% FBS and 1× GlutaMax. All cells were incubated in a humidified atmosphere of 5% CO_2_ at 37 °C. iAMs were then differentiated for nine days before the assay was performed. The differentiated iAMs exhibited spontaneous contractions at approximately 1 Hz and were capable of following pacing rates up to 6 Hz in our hands.

With exception of the determination of the optimal dose, compound treatment consisted of 5 μM of MitoTEMPO (Sigma-Aldrich, #SML0737), 5 μM of TEMPO (Sigma-Aldrich, #214000), 5 μM of TPMP (Santa Cruz Biotechnology, Dallas, TX, USA, #sc-264801), 30 μM of Sul-238 (Sulfateq, Groningen, The Netherlands), 30 μM of Vitamin E (Sigma-Aldrich, #T3251), or 30 μM of Sul-11 (Sulfateq). Compound was added to the medium at day eight of differentiation, 24 h before any effect assay. In case of tachypacing, the cells were paced using the IonOptix C-pace 100 pacer system (IonOptix, Westwood, MA, USA) and 6-well C-Dish (IonOptiX, Westwood, MA, USA) at 25 V and 5 Hz, with 10 ms pulses for 1 h at day nine. All assays were performed using passage number 15–18.

### 2.2. Drosophila Melanogaster

All *Drosophila* strains were maintained on control food consisting of 26 g/L brewer’s yeast (MP Biomedical, Irvine, CA, USA, #903312), 54 g/L D-(+)-Glucose (Sigma Aldrich, #G7021), 17 g/L agar (Boom, Meppel, The Netherlands) #76050048.5000), and 1.3 g/L Methyl 4-hydroxybenzoate (Sigma Aldrich, #H3647). *Drosophila* were kept at 25 °C on a 12 h light/12 h dark cycle during experiments. All assays were performed on prepupae.

Several experimental *Drosophila* lines were used. To assess the effect of tachypacing, W1118 V6000 wild-type *Drosophila* were used [18] (BDSC, Bloomington, IN, USA, RRID: DGGR_105924). To assess the whole body- or heart-specific effects of the chosen genes, the GAL4-UAS system was utilized. Hereto, the 4414_P{Act5C-GAL4}25FO1 (BDSC, RRID: BDSC_4414) was used to manipulate whole body gene expression, or the HandC-4.2 GAL4 line was used to manipulate heart-specific gene expression [19] (a kind gift from Prof. Dr. Achim Paululat). To assess ROS levels, the 5428_P{UAS-EGFP}8 (BDSC, RRID: BDSC_5248) [20], 67664_P{UAS-mito-roGFP2-Grx1}9 (BDSC, RRID: BDSC_67664) [21], and 67662_P{UAS-cyto-Grx1-roGFP}13 (BDSC, RRID: BDSC_67662) [22] lines were used. To assess the effect of SOD1 knockdown, the UAS lines of SOD1 KD GD (VDCR, Vienna, Austria, 31551) and SOD1 KD KK (VDCR, 108307) were used. To assess the effect of SOD2 knockdown, the UAS lines of SOD2 KD GD (VDCR, 42162) and SOD2 KD KK (VDCR, 110547) were used. To assess the effect of SOD1 overexpression, the 33605_P{UAS-Sod1}12.1 (BDSC, RRID: BDSC_33605) was used. To assess the effect of SOD2 overexpression, the 24494_P{UAS-Sod2.M}UM83 (BDSC, RRID: BDSC_24494) and 27645_P{UAS-Sod2.x}1B (BDSC, RRID: DBSC_27645) were used. GAL4 lines were crossed with UAS lines, and the F1 progeny prepupae were used for experiments.

### 2.3. Measurement of Calcium Transient for Contractility

iAMs were cultured (3.0 × 10^6^ cells per well) in a fibronectin (Sigma Aldrich, #F1141)-coated 35 mm dish in differentiation medium. Following nine days of differentiation, the cells were washed three times using differentiation medium. Subsequently, cells were incubated with 1:1000 dilution of Fluo-4 in advanced DMEM/F12 medium (488/505 nm, Thermo Fisher, #14201) for 30 min at 37 °C and 5% CO_2_. The cells were washed three times with differentiation medium, and 2.5 mL of differentiation medium was added before imaging at 20× magnification using the Leica DMi8 inverted light microscope (Leica Microsystems, Wetzlar, Germany). The cells were paced using the IonOptix C-Pace EM 6-well stimulation plate with 10 ms (IonOptix, Westwood, MA, USA), 40 V, and 1 Hz pulses to measure maximum synchronized calcium transient (CaT). Per well, 10 videos of 10 s were made. Imaging data was analyzed using the Plot Prolife function in ImageJ (version 2.0.0-rc—67/1.52i; Java 1.8.0_66 [64-bit]). Subsequently, average baseline fluorescence (F0) was subtracted from peak fluorescence (F1) to get ΔF1/F0. Experiments were independently repeated two times with twenty replicates per condition.

### 2.4. Cell Contractility Measurement

iAMs were cultured (3.0 × 10^6^ cells per well) in a fibronectin-coated 35 mm dish in differentiation medium. Following nine days of differentiation, the cells were imaged using the Leica DMi8 inverted light microscope (20× magnification). The cells were paced using the IonOptix C-pace EM, 6-well stimulation plate with 10 ms, 40 V, and 1 Hz pulses to measure maximum synchronized contractility. Traces of 10 s contraction were made using the IonWizard Software 7.8.1 (IonOptix, Westwood, MA, USA). A total of 20 cells per well were imaged. Imaging data was analyzed using the Cytosolver software 3.0.0 (IonOptix). Experiments were independently repeated two times with twenty replicates per condition.

### 2.5. Measurement of Mitochondrial Respiration

High-resolution measurement of mitochondrial respiration of the iAMs was performed using the Oroboros O2k-FluoRespirometer (Oroboros Instruments, Innsbruck, Austria). All experiments were performed with a block temperature of 37 °C, 750 rpm stirrer speed, and a data recording interval of 2 s. To assess maximal mitochondrial respiration, the SUIT-003-D012 protocol was used (https://wiki.oroboros.at/index.php/SUIT-003_O2_ce_D012; assessed on 16 October 2025). Shortly, cells were injected at a density of 2–5 × 10^5^ cells/mL. Upon injection, basal respiration was measured, followed by sequential injection of oligomycin to measure leak respiration (2.5 μM; Sigma Aldrich, #O4876), FCCP to measure maximum uncoupled respiration (average 32.5 μM as determined by titration; Sigma Aldrich, #C2920), rotenone (0.5 μM; Sigma Aldrich, #R8875), and antimycin A (2.5 μM; Sigma Aldrich, #A8674) to measure non-mitochondrial respiration. All data was analyzed using DatLab 7.4 software (Oroboros Instruments). Oxygen consumption rate was reported, non-mitochondrial respiration was subtracted, and all reads were normalized to cell number (pmol/min/cell). Experiments were independently repeated two times with four replicates for each condition.

### 2.6. Immunohistochemical Analysis of ROS Damage

iAMs were cultured (3.0 × 10^6^ cells per well) on fibronectin-coated coverslips and differentiated for nine days in differentiation medium. Following differentiation, the medium was replaced by prewarmed differentiation medium containing 250 nM of MitoTracker Deep Red (664/665 nm, Thermo Fisher, #M224626). The cells were then incubated for 30 min at 37 °C and washed three times with DPBS, followed by fixation using 4% formaldehyde (Sigma Aldrich, #P6148) for 15 min at RT. Cells were washed three times for 2 min with DPBS, after which they were permeabilized in 0.1% Triton X-100 (Sigma Aldrich, #T8787) for 10 min at RT and subsequently washed three times for 5 min with DPBS and incubated with 0.1 M Tris-HCl (pH 7.6)-buffered saline (TBS)–glycine (Sigma Aldrich, #50046) before washing three times for 5 min with DPBS and blocking with 10% goat serum (Vector Laboratories, Newark, CA, USA, #S-1000). The cells were incubated overnight at 4 °C with 4-hydroxynonenal (4-HNE) anti-rabbit antibody (1:400 in TBS-BSAc, Abcam, Cambridge, UK, #ab46545) and anti-Oxoguanine 8 (8-OxoG) anti-mouse (1:100 in TBS-BSAc, Abcam, #ab206461). The following day, the cells were washed five times for 5 min with DPBS, after which the cells were incubated for 60 min at RT with Alexa fluor 594 anti-rabbit (1:400 in TBS-BSAc, 590/617 nm, Thermo Fisher, #A-11012) and Alexa fluor 488 anti-mouse (1:200 in TBS-BSAc, 490/525 nm, Thermo Fisher, #A-11001) for 4-HNE and 8-OxoG, respectively. Finally, the cells were washed five times for 5 min with DPBS and incubated for 10 min at RT with DAPI solution (1:1000 in TBS, 359/457 nm, Sigma Aldrich, #D9564) before mounting and sealing the coverslips on a microscope slide. The resulting slides were imaged using a Leica DM6b upright microscope (Leica Microsystems, Wetzlar, Germany). To ensure similar imaging conditions for all slides, the same microscope setup was used: 40× magnification using the 40×/0.85 dry lens with 1 × 1 bin for 4-HNE and 8-OxoG imaging, respectively, and 100× magnification using the HC PL Fluotar 100×/1.23 oil lens and 1 × 1 bin for MitoTracker imaging. Imaging data was analyzed in ImageJ by measuring the intensity of fluorescence and subtracting the background intensity. Subsequent intensity was divided by the number of cells to calculate fluorescence signal per cell. For MitoTracker analysis, the image was imported in ImageJ, and auto contrast was performed. Subsequently, a 7 × 7 single-pass top-hat filter and median filter (3 × 3, single passes) were applied. The image was then made binary and analyzed using the Analyze Particles function with pixel size 0.10-infinity and circularity 0.00–1.00. Number of mitochondria was divided by the number of cells in the image. Each experiment was independently repeated two times with twenty to thirty replicates per condition. A representative image for each group was selected to illustrate the average or medium level of the group based on fluorescence intensity.

### 2.7. SOD1 and SOD2 KD in iAMs

iAMs were cultured (3.0 × 10^6^ cells per well) in a fibronectin-coated 35 mm dish in differentiation medium. On day eight of differentiation, the cells were incubated with siRNA complex. The siRNA complex consists of diluted (0.6 μM in Opti-MEM [Thermo Fisher, #31985062]) siRNA SOD1 (Thermo Fisher, #s128421), SOD2 (Thermo Fisher, #s128423), or negative control (Thermo Fisher #4390843) combined 1:1 with Lipofectamine RNAiMAX reagent (Thermo Fisher, #13778030). In each experiment, negative controls with only lipofectamine and only OptiMEM medium were also used. After 24 h, the effect of SOD1 and SOD2 KD was measured by qPCR as described below.

Primers were designed by NCBI primer BLAST (https://www.ncbi.nlm.nih.gov/tools/primer-blast/; assessed on 3 April 2025). Sequences and product length of target and reference genes can be found in Table S1. For qPCR analysis, SOD1 and SOD2 were used as target genes. Tyrosine 3-monooxygenase/tryptophan 5-monooxygenase activation protein zeta (YWHAZ), hypoxanthine phosphoribosyltransferase 1 (HPRT1), and ribosomal protein S9 (RPS9) were used as reference genes. Data were expressed as relative gene expression normalized to the reference genes.

### 2.8. Drosophila Cardiac Function

For the *Drosophila* cardiac functioning assays, W1118 *Drosophila* with or without compound treatment was used. To perform the assay, five male and five female flies were crossed in a new tube containing normal glucose (NG) diet. Three days after crossing, the adult flies were removed. For the treatment groups, with the exception of optimal concentration determination, 600 μM of MitoTEMPO, 600 μM of TPMP, 300 μM of Sul-238, or 300 μM of Sul-11 in 0.5 mL of H_2_O was added on top of the food (~4 mL) and left to dissolve.

Once the prepupae were formed, they were placed on an agar gel and tachypaced for 20 min at 20 V, 5 ms, and 5 Hz using the IonOptix C-pace EM100 pacer system (IonOptix, Westwood, MA, USA) and 4-well C-Dish (IonOptiX, Westwood, MA, USA) as previously established [23,24,25]. Following a 1 min rest, the prepupae were imaged using the inverted fluorescent phase contrast microscope (Leica DM IL LED Fluo; Leica Microsystems, Wetzlar, Germany) and a high-speed camera (DMK 33UX174, The Imaging Source, Bremen, Germany) at 10× magnification. Videos of 30 s in length were made, and heart wall traces were further analyzed using ImageJ, where a heart wall trace was made using the Z-axis profile function, as well as a kymograph using the Multi Kymograph (https://github.com/fiji/Multi_Kymograph; assessed 26 June 2023) function. Subsequently, the Z-axes profile was saved as an .xls file and analyzed further using Drosan (V3) [25] to obtain heart rate, standard deviation of interbeat interval (sdIBI), and median interbeat interval mIBI. Arrhythmia index is calculated using sdIBI/mIBI as previously described [23]. Experiments were independently repeated three times with twenty replicates for each condition.

### 2.9. ROS Measurement Drosophila

From the HandC-4.2, 67664, and 67662 lines, male and virgin female *Drosophila* were separated. Five male HandC flies were crossed with five virgin female 67664 and 67662 flies to allow heart-specific expression of mitochondrial- or cytosolic-based EGFP expression, respectively. All flies were provided NG diet. Three days after crossing, the adult flies were removed, and the eggs developed into prepupae. Once prepupae were formed, they were fixed on a microscope glass using transparent nail polish. Subsequently, the prepupae were imaged using the Leica DM6b microscope (488/510 nm). To ensure similar imaging conditions for all flies, the same microscope setting was used: magnification of 10× using the 10×/0.30 dry lens with 1 × 1 bin, 1 ms exposure time, and 3.5 gain. Intensity was analyzed using the measuring function in ImageJ, and all data were corrected for background fluorescent intensity. Experiments were independently repeated two times with three replicates for each condition. A representative image for each group was selected to illustrate the average level of the group based on fluorescence intensity.

### 2.10. Statistical Analysis

GraphPad Prism 10.2.3 (GraphPad, San Diego, CA, USA) was used for statistical analysis. Statistical details of experiments can be found in the figure legends. For data that were distributed normally, data are present as mean ± standard deviation (SD). Statistical analysis between two groups was performed using an unpaired, two-tailed Student’s t-test. Statistical analysis between multiple groups was performed using a one-way ANOVA with post hoc Dunnett’s test. A *p*-value < 0.05 was considered significant for all statistical tests.

## 3. Results

### 3.1. MitoTEMPO and Sul-238 Treatment Prevents Tachypacing-Induced Contractile Dysfunction in iAMs

**Figure 1:**
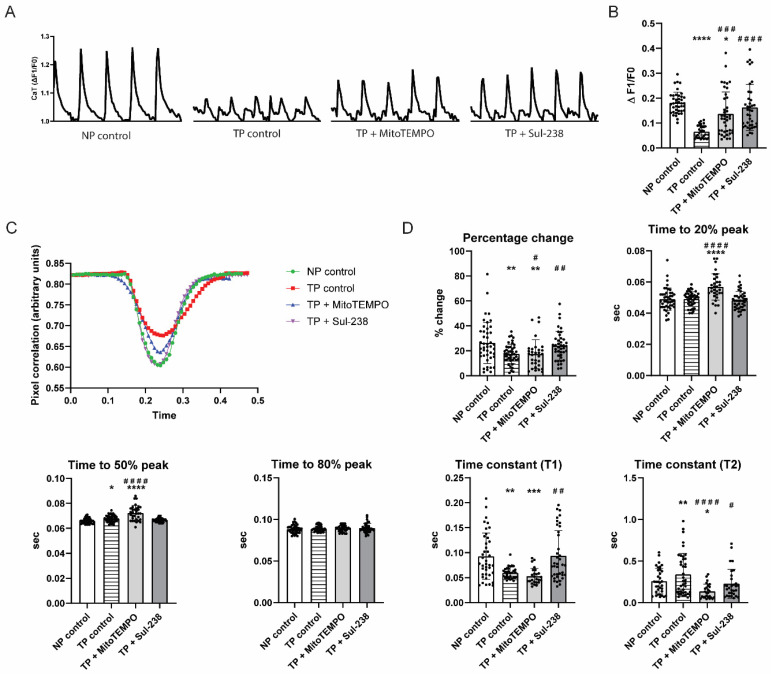
MitoTEMPO and Sul-238 treatment rescues tachypacing-induced contractile dysfunction. iAM cardiomyocytes (iAMs) were pretreated with 5 μM of MitoTEMPO (TP + MitoTEMPO), 30 μM of Sul-238 (TP + Sul-238), or water (control) and measured without pacing (NP) or after pacing (TP). (**A**) Representative traces and (**B**) quantified result of calcium transient (CaT) (*n* = 30–40 cells per condition). (**C**) Representative traces and (**D**) quantified result of contractility measurement (*n* = 30–40 cells per condition). Data are represented as mean ± SD. * *p* < 0.05, ** *p* < 0.01, *** *p* < 0.005, **** *p* < 0.001 vs. NP control by one-way ANOVA with post hoc Dunnett’s test. ^#^ *p* < 0.05, ^##^ *p* < 0.01, ^###^ *p* < 0.005, ^####^ *p* < 0.001 vs. TP control by one-way ANOVA with post hoc Dunnett’s test.

It has been recognized that tachypacing induces contractile dysfunction in several experimental in vitro cardiac cells [25,26]. However, the effect of tachypacing in iAMs has not yet been described comprehensively. We therefore first assessed the effect of tachypacing on iAMs on CaT, as well as contractility. Tachypacing of 1 h decreased CaT amplitude in iAMs significantly (Figure 1A,B), induced contractile dysfunction by decreasing the shortening of the cells (percentage change), slowed the kinetics of systolic contraction, and accelerated the kinetics of diastolic relaxation (Figure 1C,D).

Next, we tested whether pretreatment with MitoTEMPO or Sul-238, two mitochondria-targeted antioxidants, could prevent this tachypacing-induced contractile dysfunction. The optimal pretreatment dosage was first determined. For MitoTEMPO, we found a dose-dependent increase in CaT and cell shortening with increasing MitoTEMPO dosage (Supplemental Figure S1). A similar dose-dependent increase was found for Sul-238 until 30 μM. A higher dose of 50 μM of Sul-238 did not affect CaT or cell shortening after tachypacing (Supplemental Figure S2). Pretreatment with 5 μM of MitoTEMPO or with 30 μM of Sul-238 normalized tachypacing-induced CaT loss (Figure 1B). Furthermore, pretreatment with MitoTEMPO significantly increased the cell shortening and decreased early and middle systolic contraction kinetics and increased late diastolic contraction kinetics (Figure 1D), while Sul-238 treatment completely rescued cell shortening, as well as systolic and diastolic contraction kinetics (Figure 1C,D). Treatment with MitoTEMPO or Sul-238 in non-tachypaced iAMs did not affect contractile function at the applied dose, supporting the compound safety at this concentration (Supplemental Figure S3). Of note, the backbone of MitoTEMPO, TPMP, did not protect against tachypacing-induced CaT loss or contractile dysfunction as expected. However, the non-mitochondrial targeted equivalent, TEMPO, partially yet significantly improved CaT under tachypacing, although it was less potent than MitoTEMPO (Supplemental Figure S4A–D). In addition, both TPMP and TEMPO altered systolic and diastolic contraction kinetics (Supplemental Figure S4A–D). Similarly, the backbone of Sul-238, Sul-11, did not affect CaT and only showed an effect on diastolic relaxation kinetics, increasing significantly compared to the control. In contrast, the non-mitochondrial targeted equivalent, Vitamin E, significantly increased CaT, albeit less potently than the mitochondria-targeted antioxidant. Vitamin E treatment had no effect on cell shortening, slowed down mid-systolic contraction kinetics, and accelerated end-diastolic relaxation kinetics (Supplemental Figure S5A–D).

### 3.2. MitoTEMPO and Sul-238 Treatment Prevents Tachypacing-Induced Mitochondrial Dysfunction in iAMs

**Figure 2:**
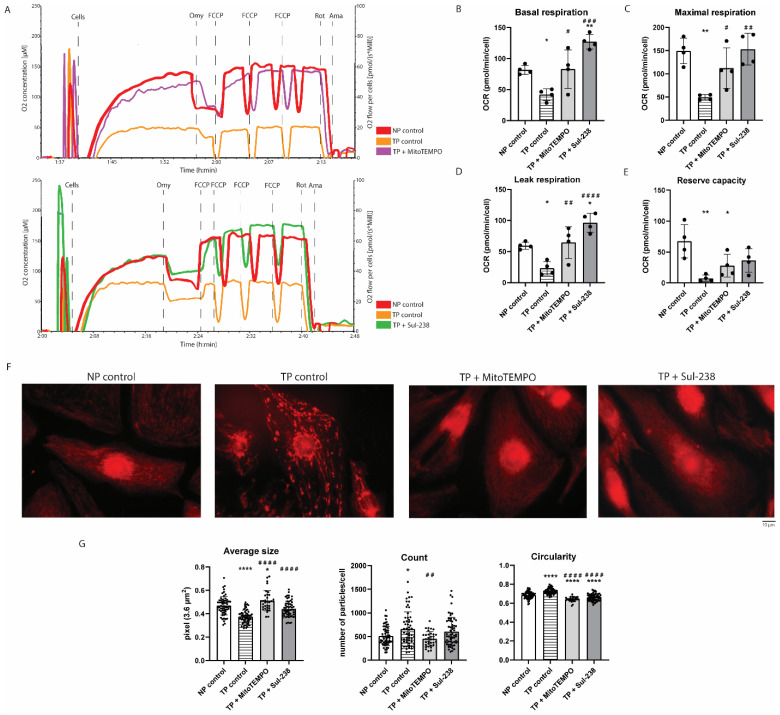
MitoTEMPO and Sul-238 treatment rescues tachypacing-induced decreased mitochondrial dysfunction. iAM cardiomyocytes (iAMs) were pretreated with 5 μM of MitoTEMPO (TP + MitoTEMPO), 30 μM of Sul-238 (TP + Sul-238), or water (control) and measured without pacing (NP) or after pacing (TP). (**A**) Representative high-resolution respirometry traces in real time under basal conditions and in response to mitochondrial inhibitors (Omy, oligomycin; FCCP; Rot, rotenone; Ama, antimycin A). (**B**–**E**) Quantification of basal (**B**), maximal (**C**), and leak (**D**) respiration and reserve capacity (**E**) (*n* = 4 technical replicates per condition). Non-mitochondrial respiration was subtracted from all data, which was subsequently normalized to cell count. (**F**) Representative images of MitoTracker staining to visualize mitochondria. (**G**) Quantified results of mitochondrial average size, count, and circularity analyzed based on MitoTracker staining (*n* = 40–60 images per condition). Data are represented as mean ± SD. * *p* < 0.05, ** *p* < 0.01, **** *p* < 0.001 vs. NP control by one-way ANOVA with post hoc Dunnett’s test. ^#^ *p* < 0.05, ^##^ *p* < 0.01, ^###^ *p* < 0.005, ^####^ *p* < 0.001 vs. TP control by one-way ANOVA with post hoc Dunnett’s test.

We found that the mitochondria-targeted antioxidants, MitoTEMPO and Sul-238, were able to prevent tachypaced-induced contractile dysfunction. Therefore, we tested their effect on mitochondrial function of tachypaced iAMs. Firstly, the effect of tachypacing on mitochondrial function was assessed using the Oroboros respirometer. Cells were exposed to tachypacing and pretreated with water (vehicle), MitoTEMPO, or Sul-238, followed by measurement of basal, leak, and maximal respiration and reserve capacity (Figure 2A). Consistently, tachypacing of iAMs resulted in a decrease in all respiratory states, as well as in reserve capacity, which was rescued by MitoTEMPO or Sul-238 treatment (Figure 2A–E).

This reduction in mitochondrial function could be due to a change in mitochondrial morphology such as fragmentation [27]. To investigate this, iAMs were stained with MitoTracker Deep Red (Figure 2F). Subsequently, mitochondrial particle size, count, and circularity were determined. Tachypacing decreased average size and increased count and circularity, indicative of functional damage due to mitochondrial fragmentation (Figure 2G). Interestingly, both MitoTEMPO and Sul-238 treatment increased average size and reduced circularity, while only MitoTEMPO decreased tachypacing-induced increase in the number of mitochondria (Figure 2G). This indicates that pretreatment with mitochondria-targeted antioxidants can prevent the functional damage, as well as mitochondrial fragmentation after tachypacing.

### 3.3. MitoTEMPO and Sul-238 Treatment Prevents Tachypacing-Induced ROS Damage in iAMs

**Figure 3:**
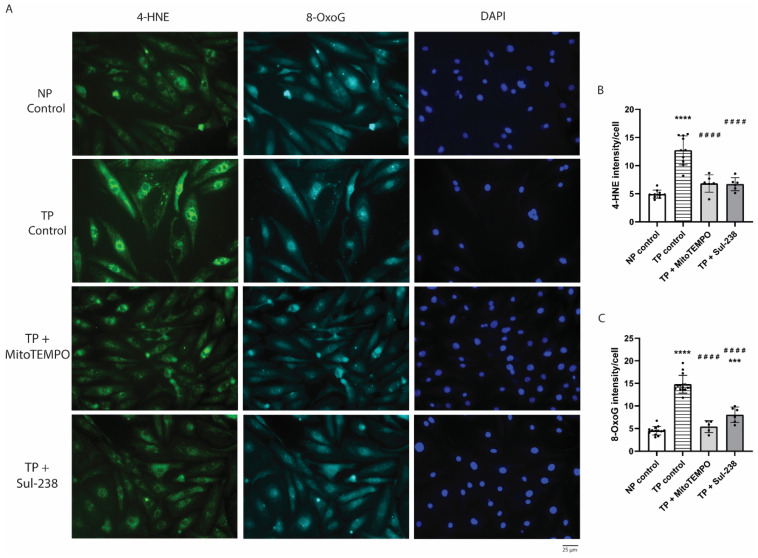
MitoTEMPO and Sul-238 treatment inhibits tachypacing-induced ROS damage. iAM cardiomyocytes (iAMs) were pretreated with 5 μM of MitoTEMPO (TP + MitoTEMPO), 30 μM of Sul-238 (TP + Sul-238), or water (control) and imaged without pacing (NP) or after pacing (TP). (**A**) Representative immunofluorescence staining of lipid peroxidation marker 4-hydroxynonenal (4-HNE) and DNA damage marker 8-oxoguine (8-OxoG). (**B**,**C**) Quantified result of 4-HNE (**B**) and 8-OxoG (**C**) intensity per cell. (*n* = 6–10 technical replicates per condition). Data are represented as mean ± SD. *** *p* < 0.005, **** *p* < 0.001 vs. NP control by one-way ANOVA with post hoc Dunnett’s test. ^####^ *p* < 0.001 vs. TP control by one-way ANOVA with post hoc Dunnett’s test.

Increased mitochondrial fragmentation and dysfunction can lead to an increase in ROS production, and in turn, ROS can damage lipids and DNA of mitochondria, generating a vicious cycle [28,29]. To test whether this is the case in our tachypaced iAMs, 4-hydroxynonenal (4-HNE), as a marker for lipid peroxidation, and anti-8-oxoguanine (8-OxoG), as a marker for ROS-induced DNA-damage intensity, were assessed (Figure 3A). Tachypacing of iAMs resulted in an increased 4-HNE, as well as 8-OxoG fluorescent intensity, indicating enhanced ROS-induced damage, i.e., oxidative stress (Figure 3A–C). Treatment with MitoTEMPO or Sul-238 completely rescued tachypacing-induced oxidative stress (Figure 3A–C). This result highlights that tachypacing induces oxidative stress, which is reversed by pretreatment with a mitochondria-targeted antioxidant (MitoTEMPO or Sul-238), suggesting tachypacing induced specifically mitochondrial oxidative stress.

### 3.4. SOD2 Knockdown Induces Contractile Dysfunction in iAMs

Since SOD1 and SOD2 are located in distinct subcellular compartments and detoxify O_2_^−^ in the cytosol/intermembrane space and in the mitochondrial matrix, respectively [30], we selected these genes to determine whether contractile dysfunction following tachypacing was specifically driven by MitoOxS rather than general oxidative stress. iAMs were transfected with siRNAs targeting cytoplasmic SOD1 or mitochondrial SOD2 to induce gene knockdown. The knockdown effect was first validated (Supplemental Figure S6A), and the effect of the knockdown process on contractile function was examined using a negative control which showed no effect on CaT or contraction (Supplemental Figure S6B–D).

**Figure 4:**
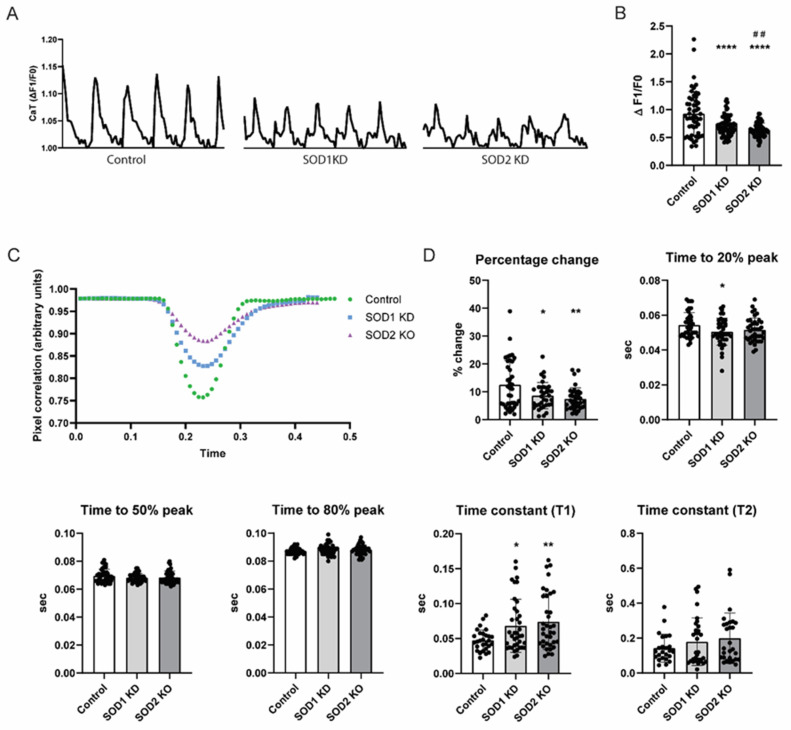
SOD2, but not SOD1 knockdown, induces contractile dysfunction in iAMs. iAMs were transfected with a lipofectamin-RNAiMAX of SOD1 (SOD1 KD), SOD2 (SOD2 KD), or water (vehicle, control). (**A**) Representative traces and (**B**) quantified result of calcium transient (CaT) normalized to control (*n* = 30–40 technical replicates per condition). (**C**) Representative traces and (**D**) quantified result of contractility measurement (*n* = 30–40 technical replicates per condition). Data are represented as mean ± SD. * *p* < 0.05, ** *p* < 0.01, **** *p* < 0.001 vs. control by one-way ANOVA with post hoc Dunnett’s test. ^##^ *p* < 0.01 vs. SOD1 KD Student’s *t*-test.

Following the knockdown, CaT and contractility were assessed (Figure 4). Both SOD1 and SOD2 knockdown decreased CaT (Figure 4A,B). However, SOD2 knockdown decreased CaT further than SOD1, indicating that MitoOxS, rather than general oxidative stress, induces a more prominent decrease in CaT. A similar pattern was observed in contractility measurement (Figure 4C,D). Here, both SOD1 and SOD2 knockdown reduced cell shortening, with the effect being more pronounced following SOD2 knockdown. The SOD1 knockdown affected early systolic contraction kinetics, whereas the SOD2 knockdown did not affect systolic contraction kinetics. Both SOD1 and SOD2 knockdown affected early diastolic relaxation kinetics, as shown by increased time constant Tau1 (Figure 4C,D). These findings strongly further underline the hypothesis that mitoOxS specifically underlies tachypacing-induced contractile dysfunction in iAMs.

### 3.5. MitoTEMPO and Sul-238 Treatment Prevents Tachypacing-Induced Arrhythmia in Drosophila

**Figure 5:**
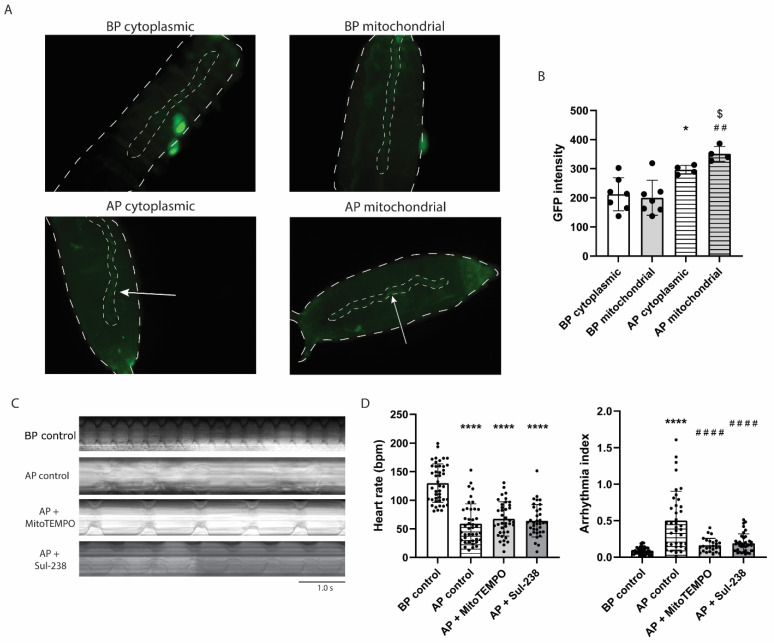
Tachypacing induces ROS production and arrhythmia in *Drosophila*, which was rescued by MitoTEMPO and Sul-238 treatment. *Drosophila* expressing a heart-specific promotor were crossed with *Drosophila* expressing a GFP-specific construct, and the F1 generation was imaged to assess cardiac-specific GFP expression in cytoplasm and mitochondria before pacing (BP cytoplasmic and BP mitochondrial, respectively) and after pacing (AP cytoplasmic and AP mitochondrial, respectively). To determine the effect of the compounds, *Drosophila* were filmed before tachypacing (BP control) and after tachypacing (AP) after pretreatment with 600 μM of MitoTEMPO (AP + MitoTEMPO), 300 μM of Sul-238 (AP + Sul-238), or water (vehicle, AP control). (**A**) Representative images and (**B**) quantified fluorescent intensity of cardiac-specific expression of GFP upon cytosolic or mitochondrial glutathione oxidation. Heart wall tube is indicated by small dotted line in the images an indicated by an arrow in the lower panels (*n* = 8 prepupae per condition). (**C**) Representative cardiac kymographs and (**D**) quantified result of the effect of tachypacing and treatment with MitoTEMPO or Sul-237 on heart rate and arrhythmia index in *Drosophila* prepupae, determined from 30 s movies (*n* = 30–40 prepupae per condition). Data are represented as mean ± SD. * *p* < 0.05, **** *p* < 0.001 vs. BP cytoplasmic or BP control by a one-way ANOVA with post hoc Dunnett’s. ^##^ *p* < 0.01, ^####^ *p* < 0.001 vs. BP mitochondrial or AP control by a one-way ANOVA with post hoc Dunnett’s test. ^$^ *p* < 0.05 vs. AP cytoplasmic by Student’s *t*-test.

To confirm our in vitro results in an in vivo model, the *Drosophila* melanogaster was employed, which is a regularly used model for studying tachypacing-induced cardiac arrhythmia [24,31,32]. To assess whether tachypacing could induce MitoOxS, male GAL4 *Drosophila* carrying a cardiac-specific promotor were crossed with virgin female UAS lines carrying a GFP-based construct for either cytoplasmic or mitochondrial glutaredoxin oxidation, which would produce green fluorescence upon oxidation [21]. The F1 progeny prepupae were used, which allowed us to assess cardiac-specific oxidation in both cytoplasm, as well as mitochondria. Before pacing, no difference was found between cytoplasmic and mitochondrial GFP intensity (Figure 5A,B). However, after pacing, both cytoplasmic and mitochondrial GFP was increased, mitochondrial GFP being even more intense than cytoplasmic GFP (Figure 5B). This result indicated tachypacing predominantly induced MitoOxS in *Drosophila*.

To determine whether tachypacing-induced increases in MitoOxS affect cardiac function, *Drosophila* were pretreated with the mitochondria-targeted antioxidants MitoTEMPO or Sul-238. As expected, tachypacing decreased heart rate and increased arrhythmia index (Figure 5C,D). The optimal doses of MitoTEMPO and Sul-238 were first determined, and we found a dose-dependent effect on the arrhythmia index of MitoTEMPO (Supplemental Figure S7A). For Sul-238, 300 μM was the optimal dose capable of significantly decreasing arrhythmia (Supplemental Figure S7C). Fully consistent with our in vitro results, both MitoTEMPO and Sul-238 treatment normalized the tachypacing-induced changes in arrhythmia index in *Drosophila* prepupae without affecting heart rate (Figure 5C,D). Of note, MitoTEMPO or Sul-238 treatment at their respective concentrations had no effect on heart rate or arrhythmia in control flies without pacing (Supplemental Figure S7A,C). Their backbone, TPMP and Sul-11, was used as a negative control and had no effect on heart rate or arrhythmia index before or after tachypacing (Supplemental Figure S7B,D), confirming it is the mitochondria-targeted antioxidant capacity that affects arrhythmia index after tachypacing. Taken together, tachypacing increased MitoOxS and cardiac arrhythmia, which were prevented by treatment with the mitochondria-targeted antioxidants.

### 3.6. SOD2 Knockdown Causes Arrhythmia in Drosophila, Whereas Its Overexpression Prevents Tachypacing-Induced Arrhythmia

**Figure 6:**
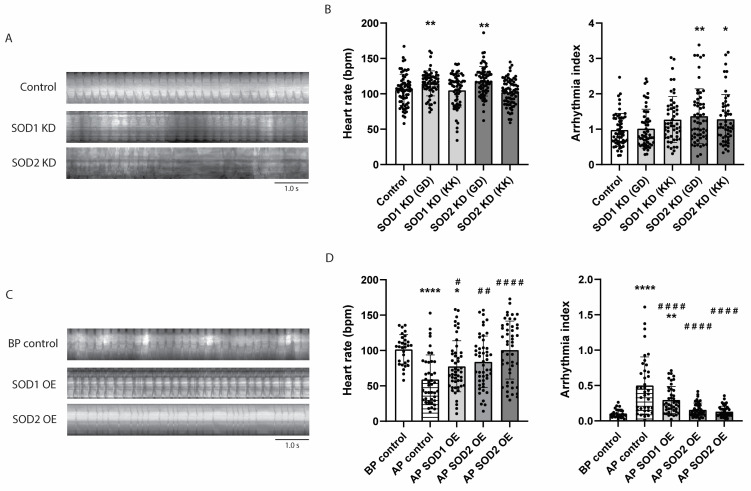
SOD2, but not SOD1 knockdown, induces arrhythmia in *Drosophila*. To assess the effect of SOD1 or SOD2 knockdown (KD), Hand-GAL4 *Drosophila* were crossed with *Drosophila* expressing siRNA of SOD1 (SOD1 KD GD or KK) or SOD2 (SOD2 KD GD or KK). To assess the effect of SOD1 or SOD2 overexpression (OE) after tachypacing, *Drosophila* prepupae were tachypaced with or without (AP control) the heart-specific expression of SOD1 (AP SOD1 OE) or SOD2 (AP SOD2 OE). Control *Drosophila* before pacing are indicated with BP control. (**A**) Representative cardiac kymographs and (**B**) quantified result of the effect of SOD1 or SOD2 KD on hearth rate and arrhythmia index in *Drosophila* prepupae, determined from 30 s movies. (*n* = 60–70 pupae per condition). (**C**) Representative cardiac kymographs and (**D**) quantified result of the effect of SOD1 or SOD2 OE on heart rate and arrhythmia index in *Drosophila* prepupae, determined from 30 s movies (*n* = 40–50 pupae per condition). Data are represented as mean ± SD. * *p* < 0.05, ** *p* < 0.01, **** *p* < 0.001 vs. control or BP control by a one-way ANOVA with post hoc Dunnett’s test. ^#^ *p* < 0.05, ^##^ *p* < 0.01, ^####^ *p* < 0.001 vs. AP control by a one-way ANOVA.

Compared to SOD1, the knockdown of SOD2 induced more severe contractile dysfunction in iAMs (Figure 4). To confirm the in vitro findings, male Hand-GAL4 *Drosophila* were crossed with virgin female UAS *Drosophila* carrying siRNA of SOD1 or SOD2 to allow a cardiac-specific SOD1 or SOD2 knockdown in their F1 progeny. Two lines were used, a GD and KK line that has a random insertion or defined insertion site of the siRNA on the chromosome, respectively. Although an increase in heart rate was found in the GD lines, this is most likely the effect of the GD insertion. SOD1 or SOD2 knockdown by itself did not affect heart rate (Figure 6A,B). Interestingly, SOD2, but not SOD1, heart-specific and whole-body knockdown increased arrhythmia (Figure 6A,B and Figure S8A), validating the in vitro finding that MitoOxS specifically induces cardiac dysfunction. Notably, tachypacing in these *Drosophila* did not further increase the effect of the knockdown, suggesting that MitoOxS acts as a downstream pathway of tachypacing (Supplemental Figure S8B).

To further confirm these findings, male *Drosophila* expressing a cardiac-specific Hand-GAL4 were crossed with virgin female UAS lines carrying a SOD1 or SOD2 gene, allowing for cardiac-specific overexpression of SOD1 or SOD2 in their F1 progeny. Following crossing, *Drosophila* prepupae were collected and tachypaced, and heart rate and arrhythmia index were determined. As observed before, tachypacing decreased heart rate, which was prevented by SOD1 or SOD2 overexpression, with SOD2 showing a better effect (31%, 42%, and 70% increase, respectively) (Figure 6C,D). This increase in heart rate was also observed in *Drosophila* prepupae without tachypacing. However, the increase found in the tachypacing model, especially that of SOD2, could not be fully ascribed to this increase in control *Drosophila* (26%, 22%, and 31% for SOD1 and SOD2, respectively) (Supplemental Figure S8C). Moreover, both SOD1 and SOD2 overexpression alleviated the tachypacing-induced increase in arrhythmia index, with SOD2 overexpression completely normalizing the arrhythmia as assessed by two independent SOD2 overexpression lines (Figure 6C,D). The overexpression by itself had no effect on arrhythmia in non-tachypaced *Drosophila* prepupae (Supplemental Figure S8C). These results suggest that decreasing MitoOxS prevents cardiac dysfunction in vivo in *Drosophila*.

## 4. Discussion

This study demonstrated that tachypacing of iAMs, as a model of AF, induces contractile dysfunction, which was accompanied by mitochondrial oxidative stress and dysfunction. These tachypacing-induced damages were completely reversed by pretreatment with a mitochondria-targeted antioxidant (MitoTEMPO or Sul-238) or by overexpression of the mitochondrial antioxidant enzyme SOD2. These results were further confirmed in an in vivo *Drosophila* melanogaster model of AF. Lastly, this study proves that MitoOxS in particular, and to a lesser extent, general oxidative stress, induces contractile dysfunction in iAMs and cardiac dysfunction in both iAMs and *Drosophila*.

In our study, the novel cell line iAM was used. This cell line originates from a single neonatal atrial cardiomyocyte clone, that is conditionally immortalized and can be differentiated. Following 9 days of differentiation, the iAMs show a stable, atrial-like phenotype with a more mature sarcomeric organization, robust action potentials, and a gene expression profile similar to primary atrial myocytes [17]. In contrast, HL-1 cells, which are often used in in vitro cardiac research, are derived from an atrial tumor and exhibit substantial heterogeneity and phenotypic drift with increasing passage number [33]. The use of iAMs compared to HL-1 cells, therefore, increases the translational potential of our results.

The findings observed in tachypaced iAMs and *Drosophila* are in line with published research on AF and other cardiac disease. In both experimental, as well as clinical models of AF and heart failure, mitochondrial dysfunction, including decreased mitochondrial respiration and aberrant mitochondrial morphology, has been observed [27,34,35]. Aberrant mitochondrial morphology in heart failure animal models and patient samples include swelling and increased fission/fragmentation [36,37,38]. The increased mitochondrial fission, in turn, can result in the decrease in mitochondrial respiration [39]. Furthermore, increased mitochondrial fragmentation was demonstrated to be necessary for ROS overproduction in cells [40]. The increase in ROS and subsequent redox signaling can, in turn, result in post-translational modifications on several key components of the mitochondrial dynamics machinery and lead to more mitochondrial fragmentation [41]. With persistent stress, damaged mitochondrial fragments may then be cleared by mitophagy, resulting in an even more depressed mitochondrial respiration, leading to activation of apoptosis, as observed in an animal model of AF [42]. We show here that our iAM and *Drosophila* models are able to recapitulate these characteristics of an AF model. Notably, genetic knockdown of the mitochondrial antioxidant enzyme SOD2 in iAMs and *Drosophila* induced MitoOxS and demonstrated that MitoOxS plays a causal role in cardiac arrhythmia and dysfunction. To our knowledge, this is the first study to establish this causal relationship.

Since oxidative stress has been implicated in the pathophysiology of AF, general antioxidants have been studied in both experimental, as well as clinical, settings. One such example is Vitamin C and E, which have been shown to be non-protective in a dog model of AF and in clinical prevention of postoperative AF [43,44]. Recently, MitoOxS has been described as a potential key driver in cardiac arrhythmia [45]. In the current study, we show that tachypacing resulted in contractile and mitochondrial dysfunction and ROS-induced damage in iAMs, while pretreatment with the established and novel mitochondria-targeted antioxidants, MitoTEMPO and Sul-238, respectively, prevented these tachypacing-induced damages. Importantly, the mitochondria-targeted antioxidants were more potent in the prevention than their general antioxidant counterparts TEMPO and Vitamin E, respectively. This result underscores the role of MitoOxS in cardiac arrhythmia. The effect of MitoOxS treatment was further confirmed in the in vivo *Drosophila* model, in which tachypacing-induced mitochondrial ROS production and arrhythmia was prevented by pretreatment with MitoTEMPO or Sul-238. This result is consistent with functional observations in other in vivo and ex vivo models of aging-induced AF [46,47]. Importantly, the use of two mitochondria-targeted antioxidants with different modes of action strengthens our conclusions by demonstrating that the protective effects are not limited to a single antioxidant mechanism.

Lastly, we validated that mitochondrial ROS specifically causes contractile dysfunction by the SOD1 or SOD2 KD in both iAMs and *Drosophila* melanogaster. This is supported in animal models showing effects on mitochondria and various aspects of cardiac diseases but not arrythmia. For instance, cardiac-specific SOD2 KD in mice was reported to reduce metabolic flexibility, reduce mitochondrial respiration, impair mitochondrial dynamics, and increase 4-HNE production within mitochondria [48,49,50]. Furthermore, SOD2 KD in animal models was reported to induce dilated cardiomyopathy by induction of apoptosis [48,49]. Interestingly, in a study by Asimakis et al., the recovery of contractile function was impaired in SOD2 KD, but not SOD1 KD, mouse hearts after ischemia reperfusion [51]. This supports our results that SOD2 KD shows increased CaT loss and contractile dysfunction compared to SOD1 in iAMS and only SOD2 KD, but not SOD1 KD, induced arrhythmia in *Drosophila*. Compared to other studies, we are the first to demonstrate that SOD2 deficiency plays a causal role in arrhythmia.

Altogether, our findings, making use of highly representative iAMs, newly show that tachypacing-induced contractile dysfunction is accompanied by mitochondrial dysfunction and ROS production in both an in vitro and in vivo model of AF. Furthermore, our study validates that MitoOxS, specifically, is essential in the development of contractile dysfunction and arrhythmia. Lastly, it highlights the potential of treatment with mitochondria-targeted antioxidant MitoTEMPO or Sul-238 for patients with AF.

Several limitations in the present study should be acknowledged. First, we made use of the novel iAM that is a physiologically relevant atrial cardiomyocyte model that resembles primary cardiomyocytes in its electrophysiological and metabolic features, unlike HL-1 cells, which do so to a lesser extent. Nevertheless, the fact that the observations are made in in vitro cells and the duration of tachypacing warrant caution regarding translational interpretation to chronic AF in patients. Similarly, the *Drosophila* has a simple, tube-like cardiac structure that can therefore not recapitulate the full complexity of mammalian cardiac morphology and function. However, *Drosophila* share conserved mitochondrial and redox pathways and thus provide a relevant model to dissect oxidative mechanisms [52]. Moreover, the fact that the iAMs and *Drosophila*, i.e., both models, showed similar findings strongly enhances the translation potential of our findings. This is enforced by the observations that multiple studies have shown that findings from cells and *Drosophila* models can be extrapolated to big mammalian models and humans [23,53,54,55]. Moreover, previous studies also reported mitochondrial dysfunction and oxidative stress in AF patients [27,56,57]. Lastly, we made use of two types of mitochondria-targeted antioxidants: MitoTEMPO, an SOD mimetic, and Sul-238, a Vitamin E derivative. Both antioxidants provided protection against tachypacing-induced mitochondrial dysfunction and cardiac arrhythmogenesis. The subtle differences in potency to normalize the measured parameters warrants further research on identifying the specific underlying pathways. Altogether, the totality of our findings highlights the high potential for relevance to human AF.

## 5. Conclusions

In this study, we show that tachypacing of iAMs induced contractile dysfunction, mitochondrial dysfunction, and ROS damage, serving as a cellular model for AF. Pretreatment with mitochondria-targeted antioxidants (MitoTEMPO or Sul-238) or overexpression of mitochondrial SOD2 prevented these tachypacing-induced adverse effects, while pretreatment with general antioxidants or overexpression of non-mitochondrial SOD1 showed less potency. These results were validated in an in vivo Drosophila model of AF. Our results underscore the therapeutic potential of mitochondria-targeted antioxidants, including MitoTEMPO and Sul-238, for patients with AF.

## Data Availability

The original contributions presented in this study are included in the article/Supplementary Materials. Further inquiries can be directed to the corresponding author.

## Supplementary Materials

The following supporting information can be downloaded at: https://www.mdpi.com/article/10.3390/antiox14121444/s1, Table S1: Sequences and product length of target and reference genes; Figure S1: Dose-dependent protective effect of MitoTEMPO on tachypacing-induced contractile dysfunction; Figure S2: Dose-dependent protective effect of Sul-238 on tachypacing-induced contractile dysfunction; Figure S3: Treatment of iAMs with MitoTEMPO and Sul-238 do not affect contractile function in non-paced cells; Figure S4: TPMP and TEMPO treatment are non- or less potent than MitoTEMPO treatment in rescuing tachypacing-induced contractile dysfunction; Figure S5: Sul-11 and Vitamin E treatment are non- or less potent than Sul-238 treatment in rescuing tachypacing-induced contractile dysfunction; Figure S6: qPCR confirms knockdown of SOD, controls of the knockdown do not induce contractile dysfunction in iAMs; Figure S7: MitoTEMPO, TPMP, Sul-238, and Sul-11 treatment do not affect heart rate and arrhythmia in non-tachypaced *Drosophila*; Figure S8: SOD2 KD on whole body increases arrhythmia in *Drosophila* whereas tachypacing does not increase arrhythmia burden in SOD cardiac-specific KD. OE of SOD does not affect arrhythmia without stressor.

## Abbreviations

The following abbreviations are used in this manuscript:

4-HNE	4-hydroxynonenal
8-OxoG	8-oxoguanine
AF	Atrial fibrillation
CaT	Calcium transient
iAMs	Immortalized atrial myocytes
MitoOxS	Mitochondrial oxidative stress
ROS	Reactive oxygen species
SOD	Superoxide dismutase
UAS	Upstream activating sequence
