# Supplementary material for: Mitochondria-Targeted Antioxidants Prevent Tachypacing-Induced Contractile Dysfunction in In Vitro Cardiomyocyte and In Vivo Drosophila Models of Atrial Fibrillation
Source: Antioxidants (Basel). 2025 Nov 30;14(12):1444. doi: 10.3390/antiox14121444 (PMC12730019; doi:10.3390/antiox14121444)
Supplement: Supplementary file 1 [file antioxidants-14-01444-s001.zip › antioxidants-3982815-supplementary.pdf]

| Gene | Sequence (5’ -> 3’) Forward primer | Sequence (5’ -> 3’) Reverse primer | Product length (bp) |
|---|---|---|---|
| *HPRT1* | CCCAGCGTCGTGATTAGTGA | TGGCCTCCCATCTCCTTCAT | 164 |
| *RPS9* | CCCTTCGAGAAATCGCGTCT | GCAGAGCGTTGCCTTCAAAC | 178 |
| *SOD1* | AAGAGAGGCATGTTGGAGACC | CGGCCAATGATGGAATGCTC | 115 |
| *SOD2* | GGTGGAGAACCCAAAGGAGAG | TGATTAGAGCAGGCGGCAAT | 173 |
| *YWHAZ* | CCCACTCCGGACACAGAATA | TGTCATCGTATCGCTCTGCC | 91 |

**Supplemental table S1. Sequences and PCR product length of target and reference genes.**

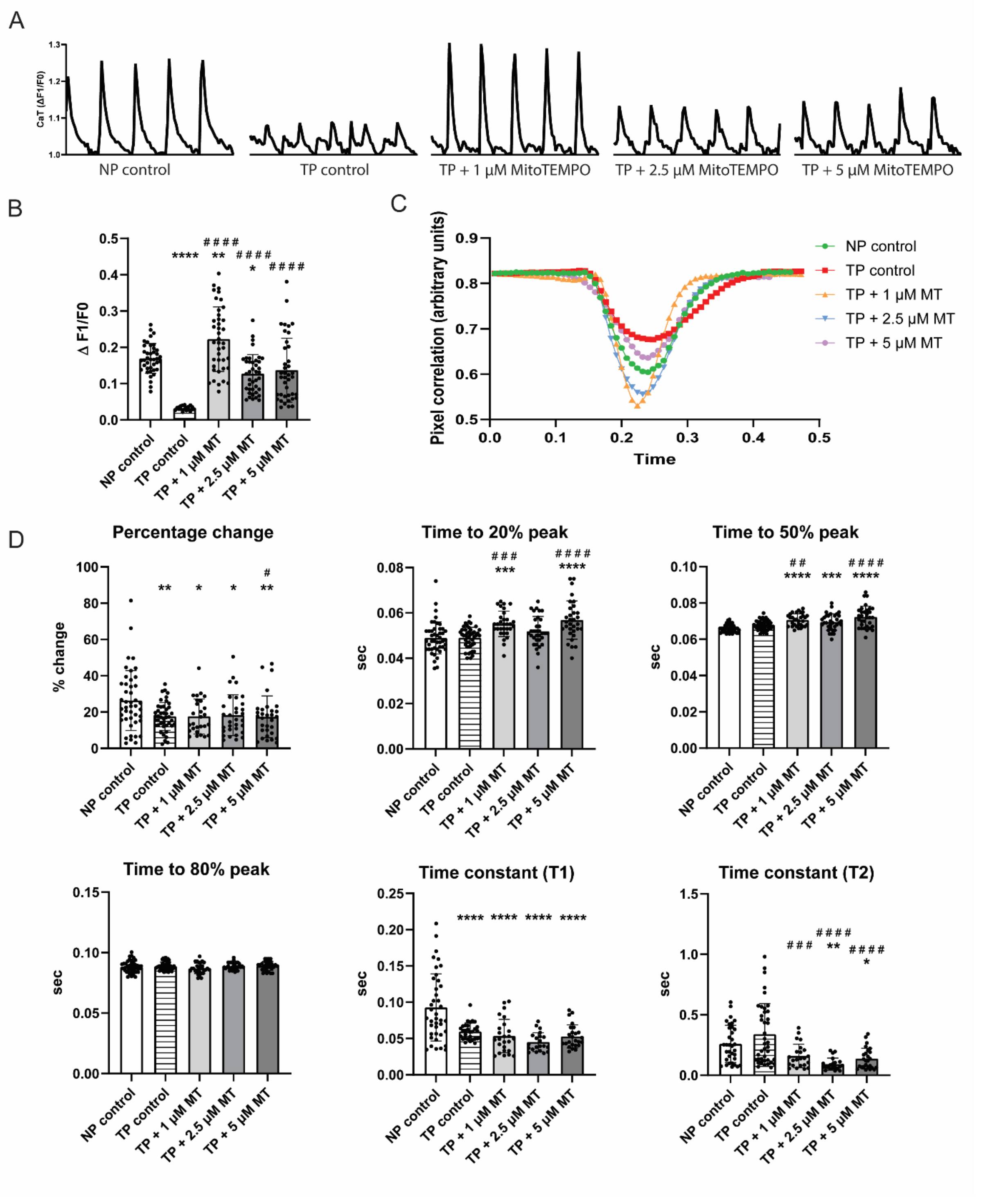


**Supplemental figure S1. Dose-dependent protective effect of MitoTEMPO on tachypacing-induced contractile dysfunction**. iAMs were pretreated with 1, 2.5 or 5 µM MitoTEMPO (TP + 1 µM MT, TP + 2.5 µM MT and TP + 5 µM MT, respectively) or water (control) and measured without (NP) or after pacing (TP). (A) Representative traces and (B) quantified result of calcium waves (n = 30-40 cells per condition). (C) Representative traces and (D) quantified result of contractility measurement (n = 30-40 cells per condition). Data are represented as mean ± SD. $^{*}P < 0.05$, $^{**}P < 0.01$, $^{***}P < 0.005$, $^{****}P < 0.0001$ vs. NP control by one-way ANOVA with post-hoc Dunnett's test. $^{\#}P < 0.05$, $^{\#\#}P < 0.01$, $^{\#\#\#}P < 0.005$, $^{\#\#\#\#}P < 0.0001$ vs. TP control by one-way ANOVA with post-hoc Dunnett's test.

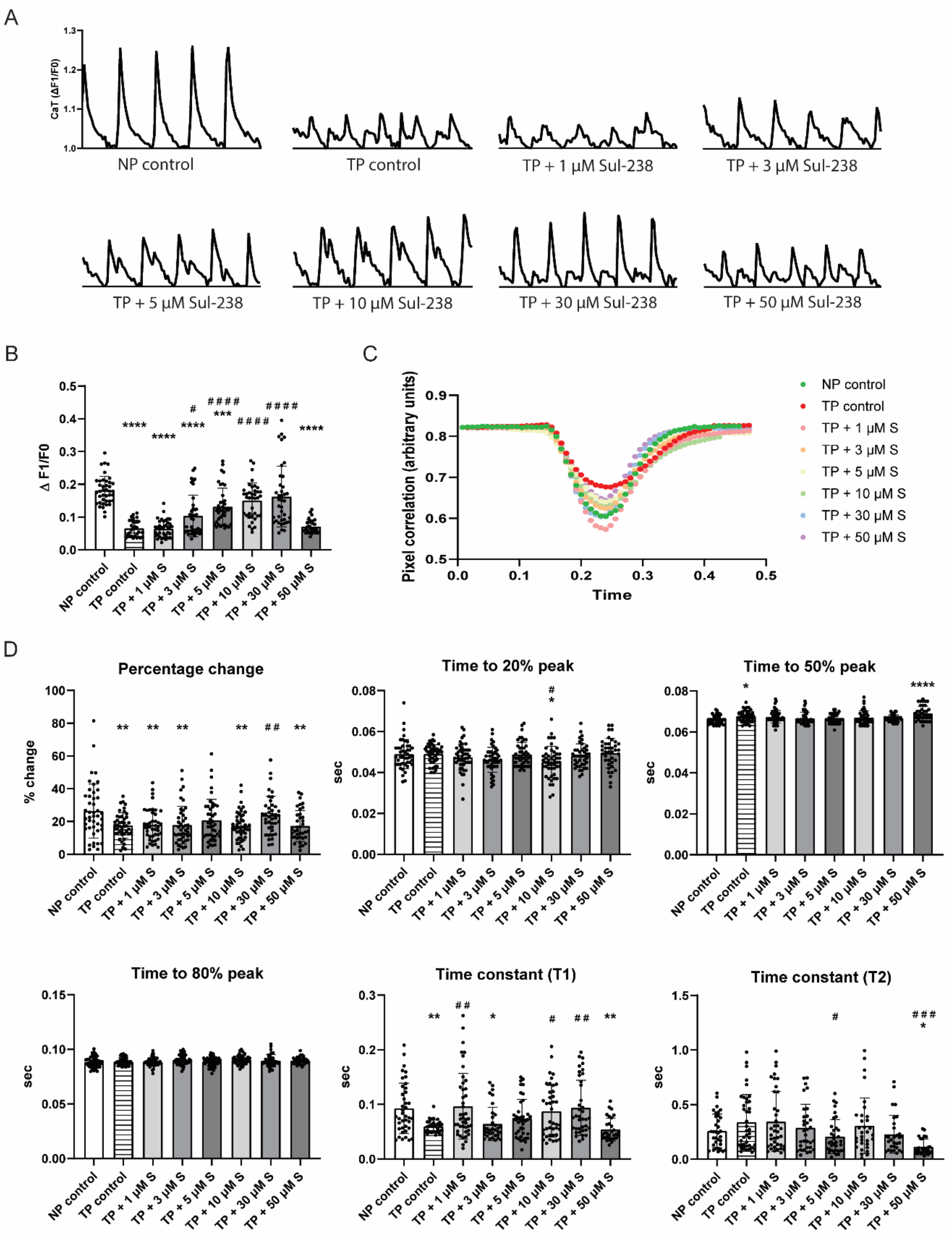


**Supplemental figure S2. Dose-dependent protective effect of Sul-238 on tachypacing-induced contractile dysfunction**. iAMs were pretreated with 1, 3, 5, 10, 30 or 50 µM Sul-238 (TP + 1 µM S, TP + 3 µM S and TP + 5 µM S, TP + 10 µM S, TP + 30 µM S, TP + 50 µM S respectively) or water (control) and measured without (NP) or after pacing (TP). (A) Representative traces and (B) quantified result of calcium waves (n = 30-40 cells per condition). (C) Representative traces and (D) quantified result of contractility measurement (n = 30-40 cells per condition). Data are represented as mean ± SD. $^{*}P < 0.05$, $^{**}P < 0.01$, $^{***}P < 0.005$, $^{****}P < 0.0001$ vs. NP control by one-way ANOVA with post-hoc Dunnett's test. $^{\#}P < 0.05$, $^{\#\#}P < 0.01$, $^{\#\#\#}P < 0.005$, $^{\#\#\#\#}P < 0.0001$ vs. TP control by one-way ANOVA with post-hoc Dunnett's test.

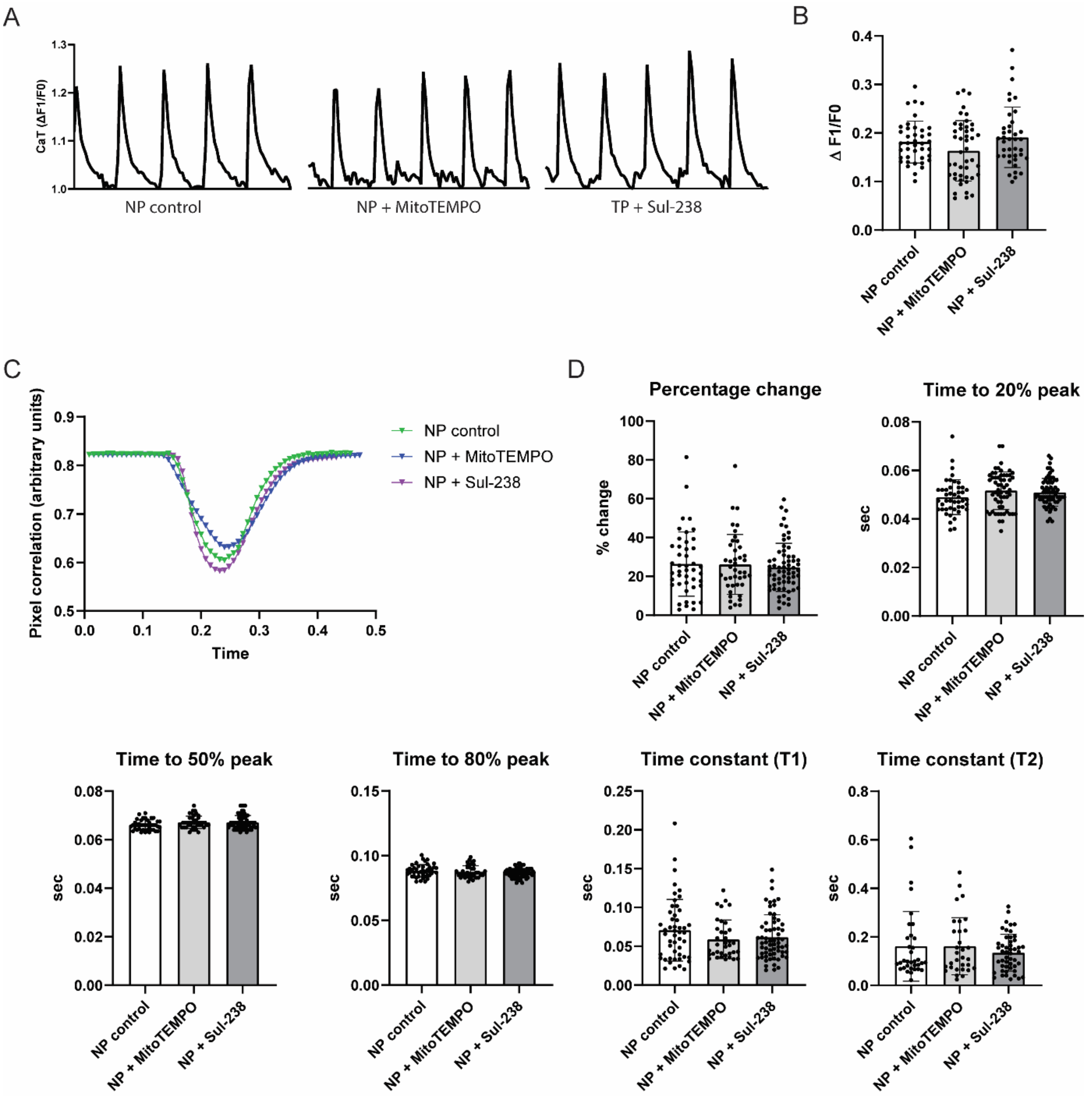


**Supplemental figure S3. Treatment of iAMs with MitoTEMPO and Sul-238 do not affect contractile function in non-paced cells.** iAMs were pretreated with 5 μM MitoTEMPO (NP + MitoTEMPO) or 30 μM Sul-238 (NP + Sul-238) or water (control) and measured without pacing (NP). (A) Representative traces and (B) quantified result of calcium waves (n = 40 cells per experiment). (C) Representative traces and (D) quantified result of contractility measurement (n = 40 cells per experiment). Data are represented as mean ± SD.

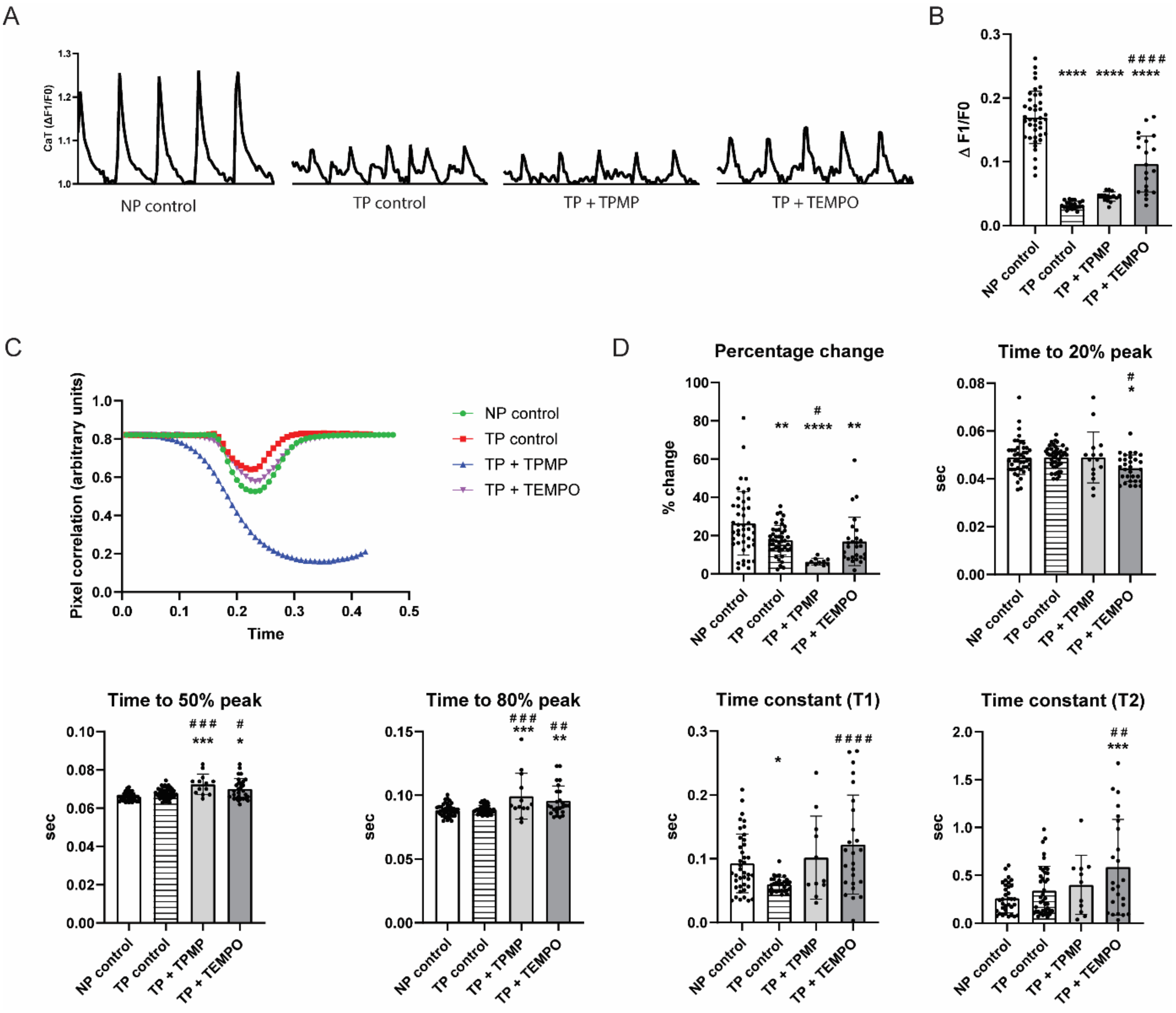


**Supplemental figure S4. TPMP and TEMPO treatment are non- or less potent than MitoTEMPO treatment in rescuing tachypacing-induced contractile dysfunction.** iAMs were pretreated with 5 μM TPMP (TP + TPMP), 5 μM TEMPO (TP + TEMPO), or water (control) and measured without (NP) or after pacing (TP). (A) Representative traces and (B) quantified result of calcium waves (n = 20-40 cells per condition). (C) Representative traces and (D) quantified result of contractility measurement. (n = 20-40 cells per condition). Data are represented as mean ± SD. $^{*}P < 0.05$, $^{**}P < 0.01$, $^{***}P < 0.005$, $^{****}P < 0.0001$ vs. NP control by one-way ANOVA with post-hoc Dunnett's test. $^{\#}P < 0.05$, $^{\#\#}P < 0.01$, $^{\#\#\#}P < 0.005$, $^{\#\#\#\#}P < 0.0001$ vs. TP control by one-way ANOVA with post-hoc Dunnett's test.

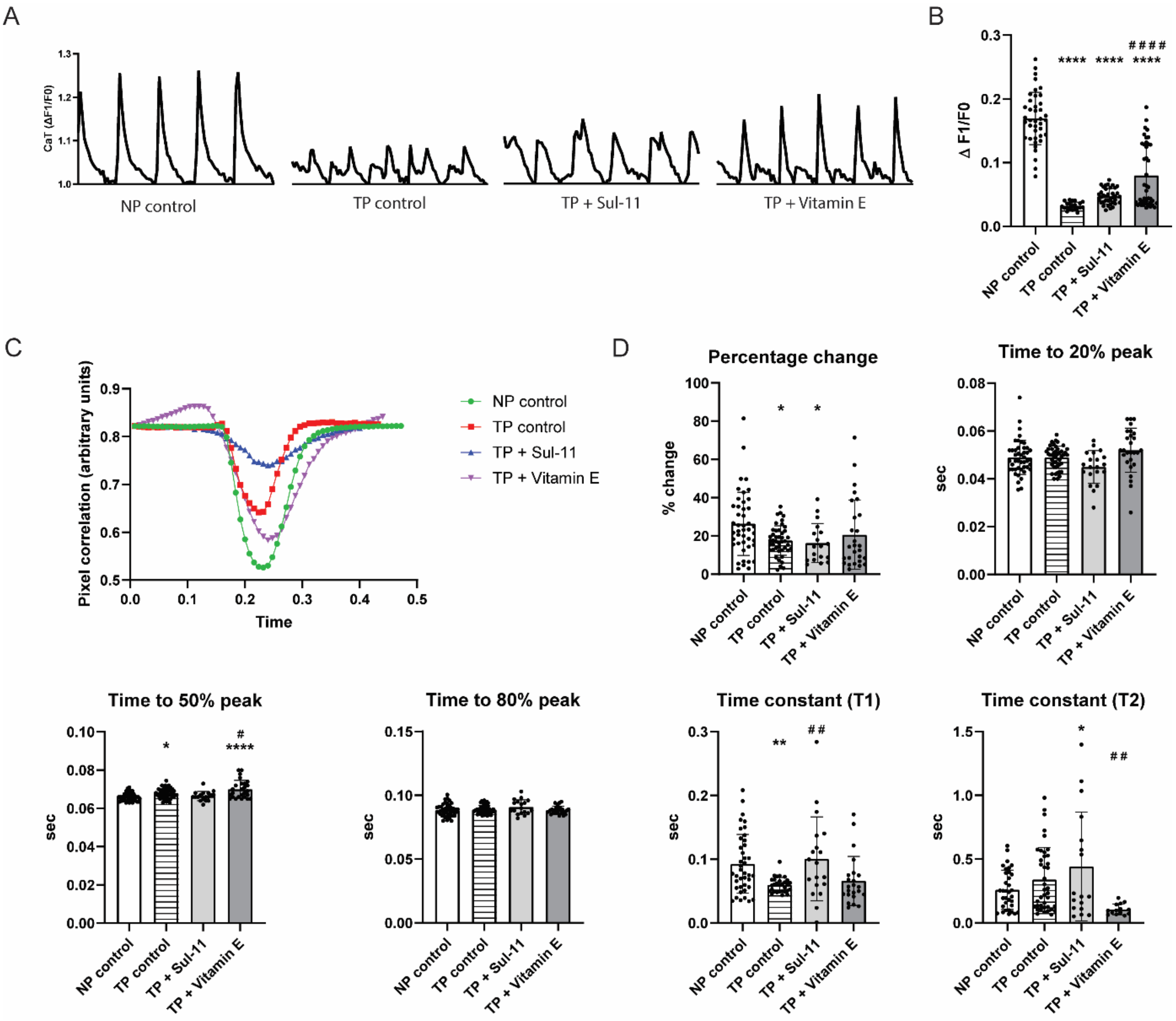


**Supplemental figure S5. Sul-11 and Vitamin E treatment are non- or less potent than Sul-238 treatment in rescuing tachypacing-induced contractile dysfunction.** iAMs were pretreated with 30 μM Sul-11 (TP + Sul-11), 30 μM Vitamin E (TP + Vitamin E), or water (control) and measured without (NP) or after pacing (TP). (A) Representative traces and (B) quantified result of calcium waves (n = 20-40 cells per condition). (C) Representative traces and (D) quantified result of contractility measurement. (n = 20-40 cells per condition). Data are represented as mean ± SD. **P* < 0.05, ***P* < 0.01, *****P* < 0.0001 vs. NP control by one-way ANOVA with post-hoc Dunnett's test. #*P* < 0.05, ##*P* < 0.01, ####*P* < 0.0001 vs. TP control by one-way ANOVA with post-hoc Dunnett's test.

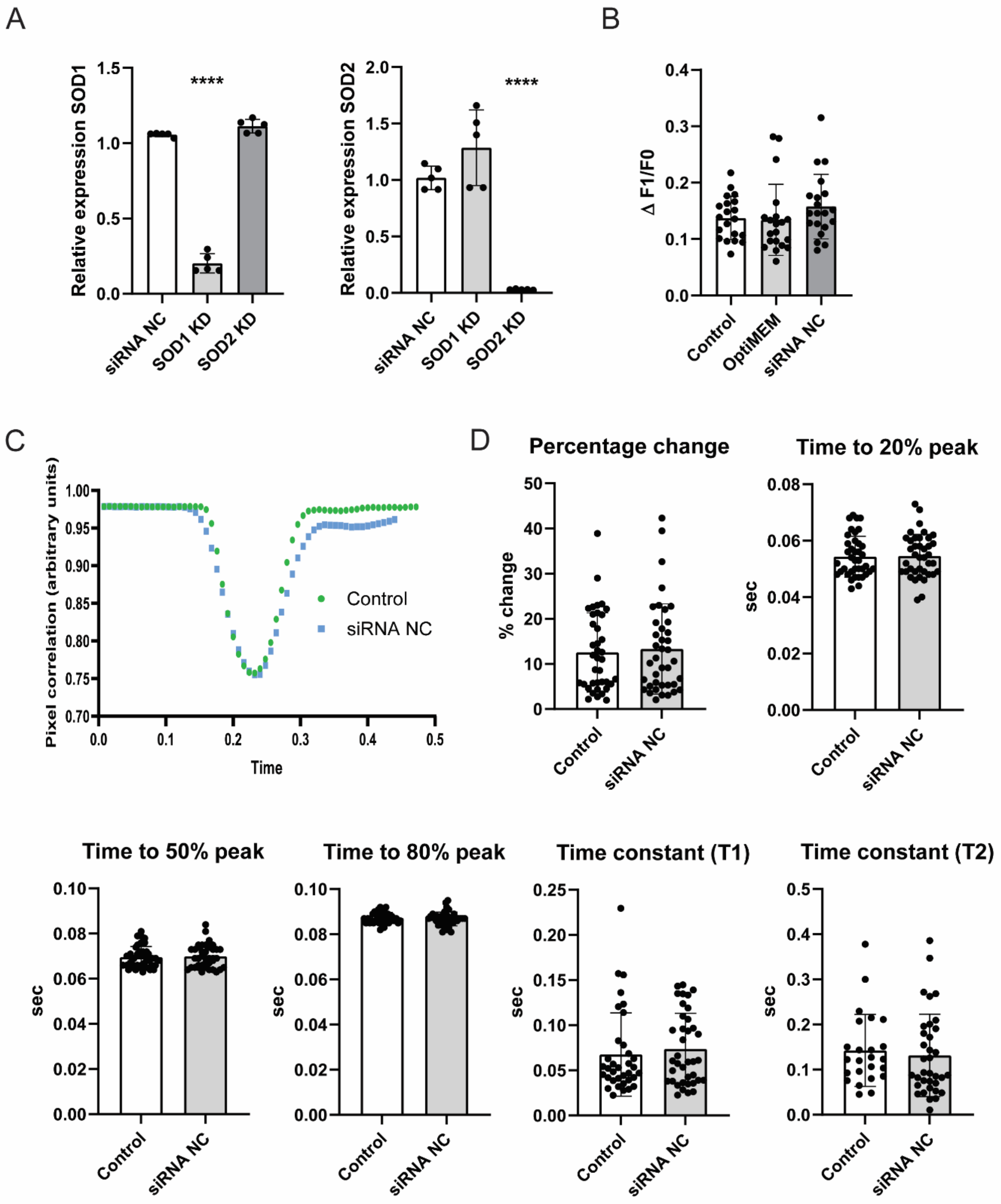


**Supplemental figure S6. qPCR confirms knockdown of SOD, controls of the knockdown do not induce contractile dysfunction in iAMs.** iAMs were transfected with a lipofectamine-RNAiMAX complex of SOD1 (SOD1 KD), SOD2 (SOD KD), an RNAi negative control (siRNA) or with water (control). (A) Relative gene expression of SOD1 and SOD2 in iAMs following knockdown of SOD1 or SOD2 (n = 5 independent experiments). (B) quantified result of calcium waves normalized to control (n = 20 cells per condition). (C) Representative traces and (D) quantified result of contractility measurement (n = 40 cells per condition). Data are represented as mean ± SD. $^{****}P < 0.0001$ vs. siRNA NC by one-way ANOVA with post-hoc Dunnett's test.

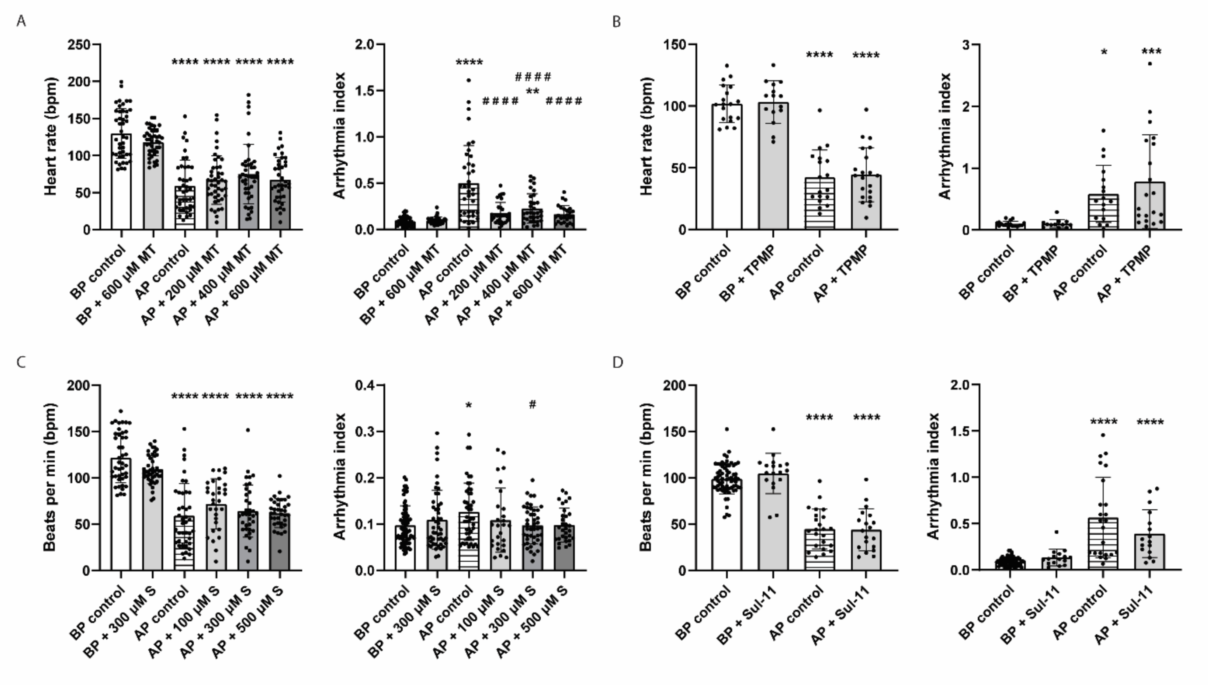


**Supplemental figure S7. MitoTEMPO, TPMP, Sul-238 and Sul-11 treatment do not affect heart rate and arrhythmia in non-tachypaced *Drosophila*.** *Drosophila* were pretreated with 600 µM MitoTEMPO (BP + 600 µM MT), 600 µM TPMP (BP + TPMP), 300 µM Sul-238 (BP + 300 µM S) or 300 µM Sul-11 (BP + Sul-11) or vehicle water (control) and filmed before tachypacing (BP) and after tachypacing (AP), a range of MitoTEMPO concentrations (AP + 200 µM MT, AP + 400 µM MT and AP + 600 µM MT), 600 µM TPMP (AP + TPMP), a range of Sul-238 concentrations (AP + 100 µM S, AP + 300 µM S and AP + 500 µM S), or 300 µM Sul-11 (AP + Sul-11). (A) Quantified result of the effect of different concentrations of MitoTEMPO on control and after-paced *Drosophila* prepupae hearth rate and arrhythmia index, determined from 30s movies. (n = 40 prepupae per condition). (B) Quantified result of the effect of TPMP on control and after-paced *Drosophila* prepupae hearth rate and arrhythmia index, determined from 30s movies. (n = 20 prepupae per condition). (C) Quantified result of the effect of different concentrations of Sul-238 on control and after-paced *Drosophila* prepupae hearth rate and arrhythmia index, determined from 30s movies. (n = 30 - 40 prepupae per condition). (D) Quantified result of the effect of Sul-11 on control and after-paced *Drosophila* prepupae hearth rate and arrhythmia index, determined from 30s movies. (n = 20 prepupae per condition). Data are represented as mean ± SD. $^{*}P < 0.05$, $^{*}P < 0.01$, $^{***}P < 0.005$, $^{****}P < 0.0001$ vs. BP control by one-way ANOVA with post-hoc Dunnett's test. $^{\#}P < 0.05$, $^{\#\#\#\#}P < 0.0001$ vs. AP control by one-way ANOVA with post-hoc Dunnett's test.

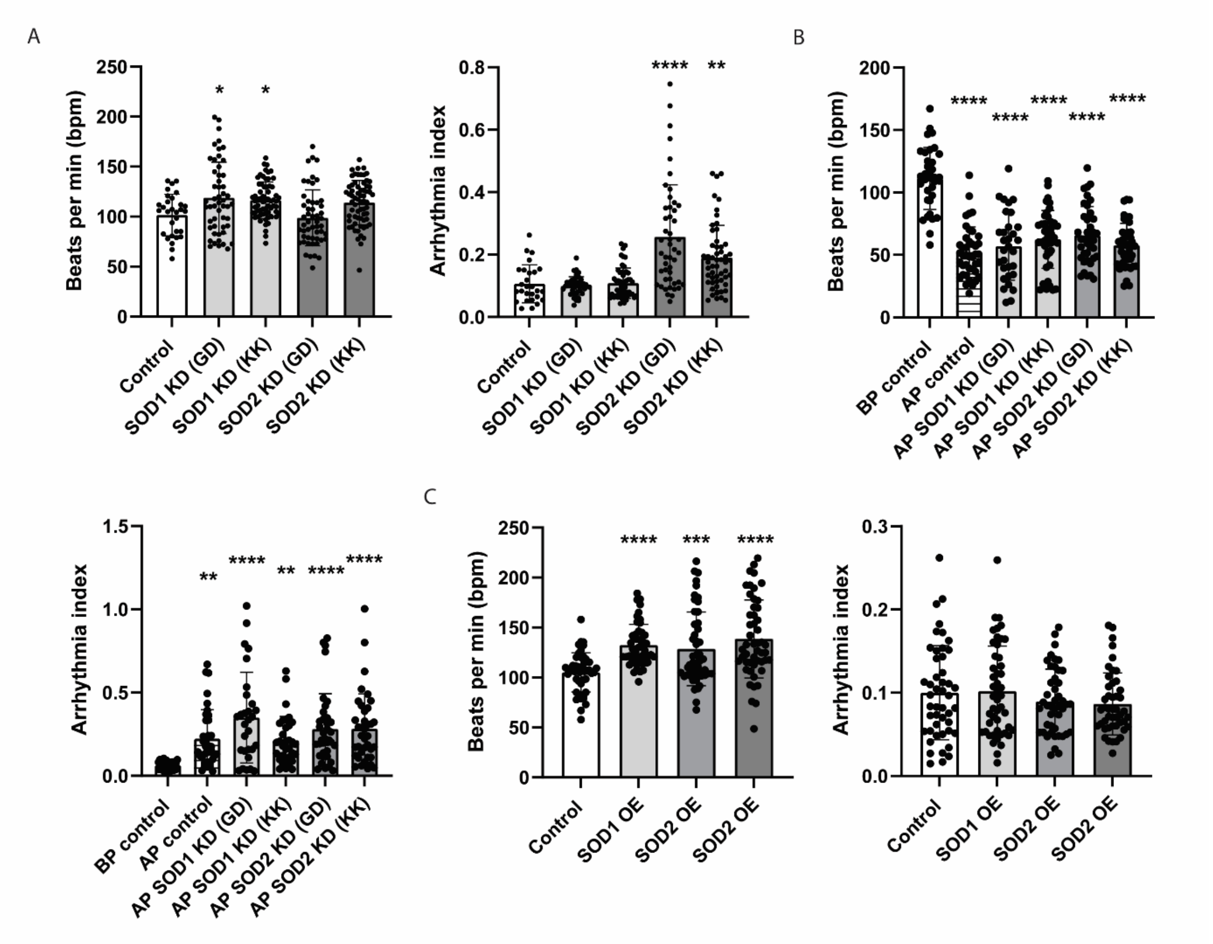


**Supplemental figure S8. SOD2 knockdown on whole body increases arrhythmia in *Drosophila* whereas tachypacing does not increase arrythmia burden in SOD cardiac-specific knockdown. Overexpression of SOD does not affect arrhythmia without stressor**. To assess the effect of SOD1 or SOD2 knockdown (KD), *Drosophila* expressing an actin whole body or heart-specific promotor were crossed with *Drosophila* expressing siRNA of SOD1 (SOD1 KD GD or KK) or SOD (SOD1 KD GD or KK). Subsequently, these *Drosophila* prepupae were imaged before (BP) or after pacing (AP). To assess the effect of SOD1 or SOD2 over-expression (OE), *Drosophila* were imaged with or without (control) the heart-specific expression of SOD1 (SOD1 OE), or SOD2 (SOD2 OE). (A) Quantified result of the effect of whole body SOD1 or SOD2 KD on hearth rhythm and arrhythmia index in *Drosophila* prepupae, determined from 30s movies. (n = 50 prepupae per condition). (B) Quantified result of the effect of cardiac specific SOD1 or SOD2 KD on hearth rhythm and arrhythmia index in *Drosophila* prepupae after pacing, determined from 30s movies. (n = 30-40 prepupae per condition). (C) Quantified result of the effect of cardiac specific SOD1 or SOD2 OE on hearth rhythm and arrhythmia index in *Drosophila* prepupae, determined from 30s movies. (n = 50 prepupae per condition). Data are represented as mean ± SD. $^{*}P < 0.05$, $^{**}P < 0.01$, $^{***}P < 0.005$, $^{****}P < 0.0001$ vs. (BP) control by one-way ANOVA with post-hoc Dunnett's test.
